# Findings and measures to eradicate methicillin resistant *Staphylococcus aureus* clonal complex 7 *spa*-type t091 in two Norwegian pig farms: a case report

**DOI:** 10.1186/s40813-021-00218-x

**Published:** 2021-06-16

**Authors:** O. M. Karlsen, K. D. Sandbu, C. A. Grøntvedt

**Affiliations:** 1 Nortura, Pb 360, Økern, 0513 Oslo, Norway; 2 Norwegian Food Safety Authority, Felles postmottak, Postboks 383, 2381 Brumunddal, Norway; 3 Norwegian Veterinary Institute, P.O. Box 64, 1431 Ås, Norway

**Keywords:** LA-MRSA, Pig farms, Eradication

## Abstract

**Background:**

The Norwegian LA-MRSA surveillance and control strategy in pig farms has been largely successful in preventing the establishment of MRSA in the pig population by identifying positive pig herds and eradicating MRSA from these. It can, however, be challenging to determine whether a particular type of MRSA is livestock-associated, particularly in cases where there is little evidence available to aid in classification.

**Case presentation:**

In two Norwegian pig farms linked by trade of live pigs, MRSA CC7 t091 was found in samples from pigs and their environment. Longitudinal sampling, with a time interval of 25 days, in one farm demonstrated an increase in samples positive for MRSA CC7 t091, supporting a classification of the finding as livestock associated. Measures to eradicate MRSA from both farms were imposed by the National Food Safety Authority. Different measures of MRSA sanitation were applied in the two farms, and MRSA was successfully eradicated from both farms.

**Conclusions:**

A high-cost, labor intensive and a lower-cost, less labor intensive MRSA eradication protocol, both including total depopulation and repopulation were successful in eradicating MRSA CC7 t091 from two case farms.

## Background

*Staphylococcus aureus* (SA) is a bacterial species with a wide range of hosts and is a significant pathogen in humans and animals in addition to being a commensal bacteria [1, 2]. Infections caused by methicillin resistant SA (MRSA) are complicated in terms of limited treatment options due to antimicrobial resistance [3]. The epidemiology of MRSA includes livestock-association MRSA (LA-MRSA), with initial reports from the Netherlands [4] and France [5] in 2005 and a subsequent study indicating high prevalence in many European pig populations [6]. LA-MRSA belonging to the clonal complex (CC) 398 has been the most widely detected LA-MRSA from livestock in Europe, particularly pig populations [7, 8]. Later other genotypes (CCs) have been added as potential LA-MRSAs [9]. Zoonotic transmission of LA-MRSA between animals and humans, and the disease-causing potential of LA-MRSA in humans is well documented [10–14].

In countries with low prevalence of MRSA in humans such as the Netherlands and Denmark, working with pigs is classified as a risk-criteria, prompting screening for MRSA upon admission to hospitals [15, 16]. In Norway however, an extensive surveillance and control strategy has been adopted since 2014 with population-wide screening for MRSA and measures imposed to eradicate LA-MRSA upon detection in pig herds [17, 18]. In humans, detection of MRSA is notifiable to the Norwegian Surveillance System for Communicable Diseases (MSIS), and detection of LA-MRSA in animals is notifiable to the Norwegian Food Safety Authority (NFSA).

In 2019 there were 2882 pig herds in Norway, 1052 holdings with sows and 1830 specialized finisher pig herds producing a total of approx. 1.63 million pigs for slaughter. During the same year the average number of sows per sow farms was 115 and the average number of weaned pigs per sow and year was 27.9 [19].

Import of live pigs to the commercial pig population in Norway is negligible [20], and previous studies have indicated introduction through MRSA positive persons as the major route of introduction of MRSA to Norwegian pig herds [21, 22]. This has led to official regulations aimed at preventing human introductions through screening of persons meeting defined risk criteria and requirements for strengthened biosecurity measures in pig farms including use of personal protective equipment (PPE) [23]. Although LA-MRSA CC398 has been the most prevalent genotype detected in Norwegian pig herds [17], other genotypes have also been detected with comparable inter-herd transmission [22]. Hence, Norway has adopted an epidemiological definition of LA-MRSA including all MRSA that persist and spread between animals in livestock holdings, not strictly limiting measures imposed to detection of certain genotypes [24].

The Norwegian LA-MRSA surveillance and control strategy in pig farms has been largely successful in identifying MRSA positive pig herds and eradicating MRSA from these. Previous studies have not found evidence of dissemination of LA-MRSA from Norwegian pig herds to the general population or the health-care sector, but has demonstrated transmission to persons occupationally exposed similar to that having been reported from other countries [11–13, 25–29].

The aim of this case report is to describe the findings of MRSA CC7 t091 in two pig farms linked by trade of live pigs in Norway, and the measures taken to follow up and successfully eradicate MRSA from these farms.

## Case presentation

Basic information about the farms and their contacts was compiled by the NFSA during the initial outbreak investigations. In addition, farm visits were made, and the herd-specific eradication plans were reviewed and discussed with the farmers. The farm veterinarians were interviewed on the phone about the health status of the pigs.

Farm A was a multiplier breeding herd. It ran a 7-, 7- and 8-weeks batch system based on a sow cycle of 22 weeks (116 days gestation, 33 days lactation and 5 days from weaning to insemination). Every batch comprised 32 sows and their offspring, and at weaning the sows were moved to the insemination unit while the litters remained in the same farrow-to-grower pens until being transported to finishing units. The farm self-recruited Norwegian Landrace maternal breeding line as the basis for their production of Topigs Norsvin 70 (TN70) hybrid gilts. All breeding was done by artificial insemination using fresh semen purchased from Norsvin. Gilts were sold as replacement stock to the central unit of a sow pool system, and the remaining growers were sold to a single finisher farm at an age of 10 to 12 weeks.

Farm B was a finisher pig herd that bought grower pigs from farm A. Farm B has three rooms each containing 15 pens with space allowance for thirteen to fifteen pigs, altogether 195 to 225 finisher pigs per room. The farm ran an all in - all out operation on room level, sending pigs for slaughter thirteen to fourteen weeks after arrival. Following the batch system of farm A, they filled an empty and clean room with pigs every seven to eight weeks.

According to herd health records, both farms had low use of antimicrobials, with treatments only initiated on medical indication and administered as individual pig treatments. In farm A, on average 2 sows/batch (6%) were treated for PPDS (mainly injectable non-steroidal anti-inflammatory drugs (NSAIDs)), and 8% of suckling piglets were treated for infectious arthritis (using IM injections benzyl penicillin procaine 60 mg/kg q 24 h for 3–7 days). Neonatal piglet diarrhea occurred on average in some to all piglets in one litter/batch, and affected animals were treated (using per os 60 mg/kg neomycin mixture q 24 h for 3 days). In farm B, 1–2% of the finishing pigs were treated for infectious arthritis or tail lesions (using IM injections of benzyl penicillin procaine 40 mg/kg q 24 h for 3–5 days or amoxicillin 7 mg/kg q 24 h for 3–5 days). Antibacterials (including zinc oxide) were not used prophylactically in either farm.

Based on notification to the NFSA of detection of MRSA in persons with contact to live pigs in the sow pool system, a wider follow-up testing of all pig farms in the sow pool system was initiated. This also included sampling the supplying multiplier breeding herd (farm A). As a multiplier breeding herd, farm A was scheduled for bi-annual MRSA testing in the surveillance program [30] with the earliest sample collection due in April, however this was rescheduled to January as a part of the contact tracing.

The initial sample collection in herd A included 5 swab cloths: three pooled swab cloths from pigs and two pooled swab cloths from the environment. In brief, the sampling was done by using sterile swab cloths (Sodibox™, Pont C’hoat 29,920 Nevez, France) soaked in sterile saline. For animal sampling, approx. 20 pigs per cloth were sampled by rubbing the skin behind both ears on each pig [31]. Environmental samples were collected by rubbing 15–20 contact points (such as drinking nipples, pen interior etc.) per swab cloth. The sampling, submission and bacteriological analysis including verification of MRSA and typing was performed as previously described (21). MRSA CC7 t091 was detected in the pooled samples of the environment of the farm and the NFSA put sanctions on the farm.

Based on the detection of MRSA in environmental samples, further sampling was performed [21] using a total of three sterile swab cloths to sample the environment and 11 cloths from pigs covering all three houses containing pigs and the different rooms in each house. MRSA was found in two of the three environmental samples and in six of the eleven samples from pigs and typing demonstrated MRSA CC7 t091.

To investigate whether MRSA CC7 t091 had the ability to persist and spread in the herd, and as such met the Norwegian criteria to be defined as LA-MRSA, herd A was resampled two times with an interval of 25 days. Sixty-eight pooled cloth samples were collected, of which 61 were pooled skin swabs of approximately 10–20 pigs per cloth and seven were pooled environmental samples with approx. 15 contact points per swab cloth. Four additional cloth samples were taken in the last sample collection: three from animals in previously unsampled groups of pigs and one from the environment. The number of MRSA positive samples was higher in the second round of sampling (Table 1).

**Table 1:** Results of longitudinal MRSA-sampling of pigs in a Norwegian pig herd (case farm A) sampled with an interval of 25 days

	Number of samples from pigs	Number of samples from the environment	Number (percent) of MRSA positive samples from pigs	Number (percent) MRSA positive samples from the environment	Number (percent) MRSA positive samples in total
First sampling	61	7	23 (38%)	2 (29%)	25 (37%)
Second sampling original sample size	61	7	29 (48%)	3 (43%)	32 (47%)
Second sampling original sample size plus four new samples	64	8	32 (50%)	3 (38%)	35 (49%)

Based on the high prevalence of positive samples and the numeric increase in prevalence from the first to second comparable sampling in farm A, it was concluded that the MRSA CC7 t091 was livestock-associated.

Farm B was sampled as a part of the contact tracing after the MRSA findings in the case multiplier herd from where it bought its grower pigs. A total of 15 pooled cloth samples from the skin of the pigs and 3 pooled cloths from the environment were collected. MRSA was detected in 4 (27%) of the pooled skin samples. Further typing demonstrated MRSA CC7 t091.

According to the control policy for LA-MRSA in pig farms in Norway, the NFSA then imposed measures to eradicate MRSA CC7 t091 in both case farms.

According to the national LA-MRSA guidelines [18], the farmers had to develop detailed plans for depopulation and subsequent measures to eradicate MRSA from the farm environment within 2 months of detection of MRSA. The farm-specific plans had to be compliant with the official LA-MRSA Guidelines [18] and approved by the NFSA. Veterinary swine health consultants employed by the slaughterhouse assisted the farmers in making these plans.

The eradication protocol for MRSA in the present case farms was based on complete herd depopulation in both farms. However, the other measures applied, were different in the two farms (Table 2). The measures taken in farm A was based on removing, discarding and renewing all internal surfaces of the pig houses. The oldest of three houses containing pigs in farm A was emptied, washed and then demolished. In farm B the pig house was washed and disinfected as it was.

**Table 2:** Measures taken to eradicate MRSA in two Norwegian pig herds

Measures taken to eradicate MRSA on farm level	Farm A	Farm B
Depopulation. All pigs slaughtered or culled.	X	X
Functional pest control programs running	X	X
The manure was collected by a local entrepreneur and applied on nearby fields before it was incorporated in the topsoil using plough and/or harrow. Tractors and equipment used for this work was washed and disinfected after the work on the case farms.	X	X
Miscellaneous, like tools, manure scrapes, boots, piglet creep heat lamps, boxes and cans, was removed from all rooms and discarded.	X	
Miscellaneous were removed from all rooms and washed and disinfected. Boots and manure scrapes were discarded.		X
Rooms were first soaked in water and then washed using water high-pressure washers to remove most of visible dirt from the floors, inventory, walls and ceilings.	X	X
Detergent was applied to all surfaces and high-pressure washers was again used to remove the rest of the visible dirt.	X	X
Internal surfaces were rinsed off with water and standing water in puddles, troughs and manure canals was drained.	X	X
Rooms were left to dry. If low temperatures and/or high humidity prolonged the time needed for drying, extra heat sources was used to dry the rooms more effectively.	X	X
All interior dismantled and discarded -Pen walls -Piglet creeps -Troughs -Feeding system -Water supply -Smoke detecting system -Slatted floors -Ventilation system -Indoor roofs with its insulation	X	
The plastic, one-piece pen divider walls were dismantled, and the bottom of the walls were filled in with acryl to prevent organic matter to build up inside the walls.		X
All rooms washed a second time	X	X
Repaired pen walls were reinstalled		X
New roofs and all new inventory/interior installed	X	
All rooms washed a third time	X	X
All surfaces disinfected using the commercial disinfectant Virocid® according to the manufacturer’s instructions	X	X
All rooms were disinfected using mobile fogging units dispersing a 1,5% Virocid® solution	X	X

**Fig. 1:**
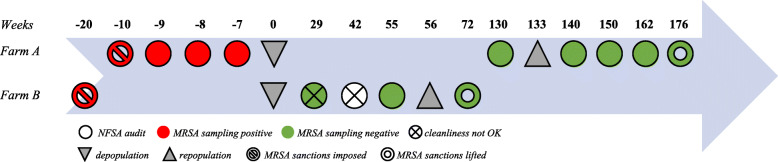
Number of weeks from first finding of MRSA to the lifting of the MRSA sanctions and the control and sampling measures undertaken by the Norwegian Food Safety A

The NFSA inspected the premises after the measures included in the LA-MRSA eradication plan had been effectuated (Fig. 1). Before restocking the farms, the environmental samples had to be MRSA negative and the NFSA had to approve the cleanliness of the farm.

The NFSA did not approve the cleanliness of farm B after inspection during week 29, especially noting a problem with organic matter seeping out from under the pen walls. This led to additional dismantling of the pen walls in farm B (Table 2). Also, during a second inspection by the NFSA in week 42, restocking was not allowed based on the finding of unsatisfactory general cleanliness. The extra rounds of washing imposed on farm B are shown in Table 2.

In farm A, pig house one was cleared for restocking in week 130 and house two in week 140. The restocking had to be from a NFSA approved MRSA negative pig farm. The negative MRSA test result on week 150, 12 weeks after the introduction of new pigs in farm A, was needed to be allowed to sell growers to NFSA approved finisher pig farms.

The post-eradication sample results for both case farms are shown in Table 3.

**Table 3:** Timeline and number of negative MRSA samples from the environment and pigs in two Norwegian pig herds after completing MRSA eradication

Timeline weeks	29	55	72	130	140	150	162	176
**Farm A environment/pigs**				13/0	9/0	3/17	4/14	2/15
**Farm B environment/pigs**	18/0	21/0	13/10					

The direct costs of the measures taken to eradicate MRSA from farm A was 10.8 million Norwegian Crowns (NOK) (approx. 1 mill EUR) and a 100.000 NOK (approx. 10.000 EUR) in farm B. The loss of revenue from pig production and the cost of purchasing replacement stock was not included in these costs.

## Discussion and conclusions

The Norwegian surveillance and control policy of LA-MRSA in the pig population is based on a socioeconomic analysis showing an economic benefit when compared to estimated costs in the human health-care sector, given a low incidence rate of LA-MRSA in Norwegian pig herds [32].

The MRSA CC7 t091 detected in the case herds was concluded to meet the epidemiological criteria for livestock-association, and the measures imposed were in accordance with national policies for LA-MRSAs.

It can be challenging to determine whether a particular type of MRSA is livestock-associated, particularly in cases where there is little evidence published to aid in classification. In such cases, the Norwegian policy dictates an epidemiological definition where evidence of between-animal transmission and persistence is sought through repeated sampling. In a previously published paper, repeated sampling after detection of MRSA CC8 t008 in a pig herd indicated very limited within-herd transmission and only focal measures with partial depopulation, washing and disinfection was adequate in eradicating MRSA from that farm [33]. One limitation of the present case report is the limited time for follow-up sampling to conclude MRSA CC7 t091 as a LA-MRSA. In any Norwegian sow farm running a seven-week batch system like farm A, the maximum time at hand to decide whether to impose LA-MRSA eradication measures or not, will be approximately 5–6 weeks because the restrictions imposed on movement of animal movement results in rapid overstocking beyond this time. A farmer will not receive any compensation for pigs culled until the NFSA imposes measures to eradicate MRSA, which will not occur before the specific genotype of MRSA has been concluded to be livestock-associated. In this case, the NFSA allowed farm A to sell growers to the already MRSA positive farm B. This allowed for the 25 days’ time interval necessary to conduct a longitudinal repeated sampling scheme for farm A.

According to the LA-MRSA Guidelines [18] pig depopulation must be concluded within 8 weeks after findings of LA-MRSA in a pig farm. The above NFSA allowance and farmer B’s acceptance to buy the rest of the MRSA positive growers and slaughter them at normal finisher pig weights, explains the 20 weeks from MRSA findings to depopulation in farm B. Farmer B normally used and continued to use PPE in his pig house during this period.

The potential consequences of a failure to act or an unsuccessful eradication of MRSA in farm A was higher than in the specialized finisher farm B, given the risk of further dissemination of MRSA to farms buying breeding stock or growers from farm A. Farm B had a newer pig barn and the farm owner was motivated for an eradication relying solely on washing and disinfection. This led to differentiated measures to eradicate MRSA from the environment in these two farms, with far more extensive measures applied for farm A.

The sample requirements to lift the NFSA restrictions were more extensive in farm A than in farm B, and this is in line with the national guidelines given that farm A was a multiplier herd and farm B was a finisher farm. After the restrictions were lifted both farms were included in the national LA-MRSA surveillance and control program [17].

High-cost MRSA eradications in pig farms puts a heavy economic burden on the farmers as the farmers co-payment for the eradication is proportional to the total cost of the measures imposed.

To maintain a robust strategy, it is important that the eradication programs are efficient both in terms of successful eradication of LA-MRSA on farm level while maintaining a cost effectiveness.

In the present case report, a high-cost, labor intensive and a lower-cost, less labor-intensive MRSA eradication program, both based on depopulation and repopulation were successful in eradicating MRSA CC7 t091 from both case farms.

## Data Availability

The data generated for the current case report are kept and stored by the corresponding author. The data are available from the corresponding author on reasonable requests.

## Abbreviations

*SA*: *Staphylococcus aureus*
MRSA: Methicillin resistant *Staphylococcus aureus*
LA-MRSA: Livestock associated MRSA
C: Clonal complex
t: *spa*-type
MSIS: Norwegian Surveillance System for Communicable Diseases
NFSA: Norwegian Food Safety Authority
PPE: Personal protective equipment
TN70: Topigs Norsvin 70
PPDS: Post-partum dysgalactiae syndrome
NSAIDs: Non-steroidal anti-inflammatory drugs
IM: Intramuscular
IU: International units
ZnO: Zinc oxide
Mg: Milligram
Kg: Kilo
Q: Every
H: Hours
